# The effectiveness of green heart application to manage modifiable risk factors of coronary artery disease in Tehran Heart Center: Study protocol for a randomized controlled trial

**DOI:** 10.1016/j.heliyon.2024.e28370

**Published:** 2024-03-22

**Authors:** Mojgan Ghavami, Saeed Sadeghian, Ayat Ahmadi, Masoumeh Lotfi-Tokaldany, Mahnaz Ashoorkhani, Fateme Haji Ali Asgari

**Affiliations:** a Cardiovascular Research Institute, Tehran Heart Center, Tehran University of Medical Sciences, Tehran, Iran; b Knowledge Utilization Research Center, Tehran University of Medical Sciences, Tehran, Iran; c Department of Health Education and Promotion, School of Public Health, Tehran University of Medical Sciences, Tehran, Iran; d Department of Information Technology, Virtual School, Tehran University of Medical Sciences, Tehran, Iran

**Keywords:** mHealth, Application, Coronary artery disease, Risk factor, Prevention

## Abstract

The burden of cardiovascular disease (CVD) is diminishing in developed countries. However, in middle- and low-income countries the CVD death rates are growing. CVD is the most common cause of death and disability in Iran and accounts for nearly half of all mortalities in Iranians. Therefore, preventive strategies by risk factor modification are a top priority in the country.

Recently, Mobile-Health (mHealth) technology has been the focus of increasing interest in improving the delivery of cardiovascular prevention, targeting a combination of modifiable risk factors.

This parallel-group single-blinded randomized controlled trial study has been designed to evaluate the impact of using a mHealth application on risk factors control. Individuals aged between 25 and 75 years who have documented CVD by coronary angiography in Tehran Heart Center and have at least one uncontrolled risk factor from the three including hypertension, dyslipidemia, and current cigarette smoking will be included. We are going to randomize 1544 patients into two study arms as follows: 1- Intervention: usual care + mHealth 2- Control: usual care + paper-based recommendations and educational materials. After 3 and 6 months of follow-up, the status of risk factors will be determined through outpatient visits and face-to-face interviews for both arms. Outcome: Successful risk factor control will be measured after 3 and 6 months.

Nowadays, mHealth is becoming increasingly popular, providing a good opportunity for constant monitoring of risk factors and changing health behavior in a target population. Meanwhile, providing evidence for the effectiveness of health intervention delivery using mobile technologies could help health providers encourage their at-risk population to stop smoking, control blood pressure and blood cholesterol, and participate in regular physical activity. While the burden of CVD is growing in developing countries, this type of intervention can be a cost-effective way to reduce it in these countries.

## 1 Introduction

Cardiovascular diseases (CVD) remain a significant health concern, especially in middle- and low-income countries like Iran where the mortality rate from CVD is on the rise, despite a decreasing trend in developed countries [1,2]. Preventive strategies have played a crucial role in reducing coronary artery disease (CAD)-related mortality in Europe and the United States, emphasizing the importance of focusing on individual and population-level risk factors [3,4].

In Iran, CVDs are a leading cause of death and disability, accounting for nearly half of all annual deaths [5]. With a large young population transitioning into high-risk CVD age groups in the future, the potential impact on mortality and disability is concerning, especially among productive forces under 55 years old [[6], [7], [8], [9]]. The burden of CVD and its associated risk factors, including cigarette smoking (CS), hypertension (HTN), dyslipidemia (DLP), diabetes, obesity, and inadequate physical activity, poses a significant challenge for healthcare systems in developing countries. Targeted preventive strategies addressing these risk factors and promoting healthy behaviors are essential to reduce mortality and disability from CVD [10]. Given the need for innovative approaches to optimize secondary prevention and treatment goals, Mobile-Health (mHealth) technology has emerged as a promising tool to strengthen preventive efforts in cardiovascular health [11]. Leveraging mobile phone applications or “apps” is a suitable and accessible method for delivering interventions to effectively manage CVD risk factors [12,13].

According to recent literature reviews, mobile-based health interventions have a strong positive effect on lifestyle modification and prompting physical activity and may be used as an adjuvant method among patients with CVD for secondary prevention [14,15].

This manuscript introduces a study on the use of mHealth technology to assist patients with CAD in controlling three key CVD risk factors HTN, DLP, and CS. By developing evidence-based interventions guided by clinical guidelines, this study aims to evaluate the effectiveness of this approach in managing these risk factors in the Iranian population. This research underscores the importance of adopting effective preventive strategies that are widely applicable and implementable to address the increasing burden of CVD in diverse populations.

## 2 Materials and method

### 2.1 Ethical considerations and registration

Written Informed consent was obtained from each patient before recruitment and the study protocol conforms to the ethical guidelines of the 1975 Declaration of Helsinki as reflected in a priori approval by the institution's human research committee. The study was approved by ethical committees of the Tehran University of Medical Sciences (Ethical code: IR. TUMS.MEDICINE.REC.1399.032). This study was registered in the Iran Randomized Clinical Trial Center with the ID of IRCT20221016056204N1 on March 12, 2023.

## 2.2 Funding

The source of funding is the National Institute for Medical Research Development (NIMAD) with grant number 4001391 with no role in study design, data collection, analysis, interpretation, writing, and submission of the manuscript.

### 2.3 Design

This parallel-group single-blinded randomized controlled trial study, has been designed to compare the impact of 6 months using an mHealth application and paper-based educational content on risk factors control in CAD patients.

The study protocol is according to the Standard Protocol Items: Recommendations for Interventional Trials (SPIRIT) 2013 Statement [16].

### 2.4 Study setting

Tehran Heart Center (THC) is a major academic tertiary-care hospital specializing in cardiovascular disorders in Iran which is affiliated with Tehran University of Medical Sciences with 460 inpatient beds, 11 operating rooms, and 6 active Cath Labs [17]. We will include eligible individuals who have attended the THC for diagnostic and treatment services. Patients should have a history of CAD defined as myocardial infarction, percutaneous coronary intervention, coronary artery bypass graft surgery, or coronary angiography confirming stenosis >50% in at least one of the major epicardial coronary arteries or their main branches in the last 6 months, along with current uncontrolled HTN, uncontrolled DLP, or current CS (The definitions of uncontrolled risk factors are described later in “Definition” section). The diagnosis of CAD is made by expert cardiologists. Given that the application is designed to control risk factors of CAD, individuals entering the study are required to have at least one uncontrolled risk factor to assess the efficacy of the app in improving these factors. Individuals over the age of 75 will not be included in the study due to the potential challenges associated with utilizing mobile technology, as well as individuals under 25 due to their lower likelihood of presenting with CAD. Regarding the 6-month follow-up period, patients who are willing to remain in the study for the entire duration will be included. Considering the potential difficulty of utilizing the application in some older individuals, especially those with low literacy levels, the study sample will be limited to individuals who have the ability and willingness to use the application, either independently or with assistance from a trusted relative they lived with. Due to the difficulty of physical presence in patients with significant morbidity and chronic illnesses, and the challenges in obtaining informed consent from individuals with cognitive impairment, they will be excluded from the study. The mentioned conditions are determined by medical history and/or physician diagnosis.

The details of inclusion and exclusion criteria are listed in Table 1.

**Table 1:** Eligibility criteria for the participants.

Inclusion criteria	Exclusion criteria
▪ History of coronary artery disease^a^ ▪ Having at least one uncontrolled risk factor^b^ ▪ Aged between 25 and 75 years ▪ Inclination to stay in the study for 6 months ▪ The participant's (or one of their trustworthy relatives who live with them) having and ability to use a smartphone ▪ Willing to control the risk factors using an application ▪ Provides informed consent to participate in the study	• Mental retardation • Individuals with prohibitive morbidities that require close face-to-face monitoring. • End-stage chronic disease such as hepatic and/or renal failure, heart failure, active cancer

a History of myocardial infarction, percutaneous coronary intervention, coronary artery bypass graft surgery, coronary angiography confirming stenosis>50% in at least one of the major epicardial coronary arteries or their main branches.

b Including uncontrolled hypertension, uncontrolled dyslipidemia, and current cigarette smoking for ≥12 months.

## 3 Intervention

### 3.1 Intervention and control groups

Hypertensive participants in both arms will be educated on the correct Blood Pressure (BP) measurement method at the baseline face-to-face visit. In the next (3 and 6 months since the randomization) visits, patients in the control group will be asked to bring the paper BP chart of one-week daily home measurements for documentation.

Smoking cessation pamphlets and booklets designed by THC will be presented to smoker patients in the control arm. All patients in the control arm also will receive paper-based lifestyle modification recommendations including a healthy diet and physical activity schedule.

### 3.2 Green heart application

The application named “**Green Heart**” can provide individualized treatment for each risk factor according to the current international guidelines. Furthermore, mHealth will supply knowledge for an accurate method of home BP measurement. Thereafter it averages one-week recorded BP and recommends the patient visit the doctor or take drugs at an appropriate time and regulates the time intervals of recommendations concerning the results. Additionally, this app is going to advise about lifestyle modification including low-salt, low-fat, low-sugar, and high-fiber diets, increasing the level of physical activity, weight loss, and quitting smoking. One of our important goals is smoking cessation. To achieve this goal, we have designed an individualized algorithm according to each person's status regarding the level of his/her inclination to smoke, amount of consumption, etc. We are going to reduce the temptation even in patients who will quit smoking by close follow-up. The contents of messages will be approved by related specialists in each section. To develop the application, we will follow 4 steps.

1) Planning: Providing and extracting the evidence from the American College of Cardiology/American Heart Association (ACC/AHA), the European Society of Cardiology (ESC), and the National Institute for Care and Excellence (NICE) guidelines and adapting them for the Iranian population by consulting with experts.
2) Analysis and designing: Determining the rules for judging criteria and designing the algorithmic diagram for each risk factor.
3) Implementation: Installation of database and designed algorithms through coding by an Information Technology (IT) specialist. Data expert software programming will be implemented in two phases. 1-Build a smart short message service robot to interact with target people by sending motivational and inhibitory texts to evaluate the extent of patient coverage; 2- Build a more comprehensive app using the results from step one.
4) Evaluation: Revising the app by experts and reflecting on the revisions. To examine the app, initially, we will apply it to our research center personnel and get their feedback. If necessary, we will revise it in the pilot phase. The installation of the software app will be performed on mobile devices of eligible participants by our team. Participants will also be taught how to use the app. To resolve the problems, the patients will be asked to work with this app in the presence of our staff. To respond to any existing questions, we will also give them a telephone number for technical support. Connecting to the internet is not needed during the process of answering questions, and receiving advice and reminders. Whenever the person connects to the internet, the data will be collected and saved to the server.

#### 3.2.1 Use of guidelines in the development of the app

When developing the app, we will incorporate the following guidelines into the algorithm of the app.

• ESC Guidelines on prevention of DLP [18].
• ACC/AHA Guideline on arterial HTN [19].
• NICE smoking cessation guideline [20].

These guidelines will be converted into computer-interpretable functions and applied to assessment and feedback systems.

#### 3.2.2 How does the green heart app work?

1. Providing condition-specific education, presenting easily-understandable information, customized to each patient, and enforcing behavior change.
2. Providing education using different modalities (multimedia, text, interactive material) to reinforce teaching.
3. Providing a means of continuous feedback to find out what the patient already knows,
And what they have learned and correct any misinformation.
4. If applicable, track progress towards goals, for example in the case of smoking cessation support.

#### 3.2.3 Contents of the green heart app

The app will consist of 3 modules, each designed to address a specific risk factor.

1 Smoking
2. HTN
3. DLP

### 3.3 The module of the app which targets smoking cessation includes the following features

• Questionnaires to track the status of the patient: Baseline and follow-up forms (assessment of the degree of nicotine dependence by Fagerström test [21], the duration of smoking, the frequency of quitting, the daily cost of smoking, the use of hookah, the state of the desire to smoke, and the reason for the desire to smoke)
• Stream of educational content on the risks of smoking, benefits of quitting, techniques and tips on how to cope with cravings and withdrawal
• Stream of supporting messages: Motivational messages (e.g., The effect of quitting on improving health and the amount of recovered cost in case of complete smoking cessation), encouraging, and warning messages
• Quit plan builder: The personalized quit plan builder, Automated messages and feedback to help the patient follow the plan, a Visual timeline for viewing progress
• Features to increase adherence to the app include an easy tutorial to familiarize the patient with the main app features and push notifications to keep the patients in touch with the app even when the app is closed.

### 3.4 The module of the app which targets HTN includes the following features

• Educational information to improve knowledge of HTN: Offering info about the risks of consequences of uncontrolled HTN and benefits of managing HTN, selecting an appropriate sphygmomanometer, guidance to choosing the size of the BP cuff according to the size of the arm circumference and standard technique of measuring BP
• Educational information to improve lifestyle: Offering info on how lifestyle changes can help lower BP and reduce the risk of heart disease
• Support for Self-monitoring of BP: Patients log their BP twice a day, seven days a week (using an input form with the possibility for refresh/reinsertion of information) according to the guidance of entering systolic and diastolic BP based on the monitor screen of the digital device, App keeps track of systolic and diastolic BP and shows trends with graphs and charts. Patients can view trends of their BP over time. Automatic feedback on the entered values by using color codes for specific ranges and showing alerts is provided.
• Reminder functionality for improving medication adherence: Setting reminders to take medications at a specific time.

### 3.5 The module of the app which targets DLP includes the following features

• The educational content on the subject of DLP: Info about the risks and consequences of uncontrolled DLP and benefits of managing DLP
• Educational content for a healthy lifestyle: Info on how a healthy lifestyle can help reduce lipid levels and decrease the risk of heart disease and heart attacks
• Reminder functionality for improving medication adherence: Setting reminders to take medications at a specific time
• Support for self-monitoring of Low-density Lipoprotein Cholesterol (LDL-C): A space for entering the number of LDL-C according to the picture guide based on the patient's lab test sheet is provided. After the user enters the LDL-C, questions about the type of DLP medication (statin/ezetimibe) and its dose will be asked, and if the entered number is higher than the target, a corresponding message will be sent to him/her about the necessity of referring to the doctor for increasing the dose of the medication or to add another category of medication and if it is normal, it is recommended to continue the drug and do a lab test again after 12 months.

#### 3.5.1 How the patients should use it?

To utilize the app, participants need to have a smartphone and internet access, although the final product may include some offline features as well. Patients consume teaching material and send feedback. Ideally, the user will open and use the program several times a day.

#### 3.5.2 How to measure application adherence?

To measure patient engagement, we're going to assess both frequencies and the intensity of app usage: The frequency of app usage will be measured by logging “system-usage” data (the number of logins will be recorded per participant). The intensity of usage will be measured by recording the number of viewed pages and several filled-out self-report questionnaires.

### 3.6 Study outcomes

**Primary outcome:** Successful risk factors’ control measured at 3 and 6 months after randomization.

This outcome will be categorical as follows.

1 No controlled risk factors
2 One controlled risk factor
3 Two controlled risk factors
4 Three controlled risk factors

**Secondary outcomes**: Includes risk factors control in each level of stratification (7 levels presented early in the method section).

1 Number of participants who remain active users of the app till to the end of the intervention.
2 Controlled CS
3 Controlled HTN
4 Controlled DLP
5 Controlled CS + HTN
6 Controlled CS + DLP
7 Controlled HTN + DLP
8 Controlled HTN + DLP + CS

The definition of being controlled for each risk factor is presented in the “Definitions” section. Each risk factor that meets the outcome will be regarded as a successful intervention.

### 3.7 Definitions

• **Current cigarette smoker:** Average of ≥5 cigarettes daily use in at least the year before enrollment.
• **Successful smoking cessation:** Continuous abstinence in at least 4 weeks during 6 months following up.
• **Uncontrolled DLP:** Description for uncontrolled DLP will be based on the serum level of LDL-C which depends on the concomitant comorbidities including diabetes mellitus and CVD [18]. The related cut-off value for the serum level of target LDL-C is ≤ 55 mg/dL. Values more than the listed cut-off value are considered uncontrolled DLP.
• **Controlled DLP**: Lowering serum level of LDL-C by < 55 mg/dL and ≥50% reduction from baseline in any of the follow-up visits at the end of 3 and 6 months from randomization and remaining controlled in the next visits during the study.
• **Uncontrolled HTN:** A patient who has a history of HTN with/without using the antihypertensive drug is considered hypertensive. Participants will be considered as having uncontrolled HTN according to the ACC/AHA guidelines [19].
• **Controlled HTN:** Maintaining BP below the target treatment level (130/80 mmHg) at the time of office measurements (average of 2 readings obtained on 2 separate occasions with proper patient preparation and appropriate technique) at the end of 3 and 6 months from randomization and/or during one-week daily home measurements (average of records) before clinic visits.

### 3.8 Participants

According to eligibility criteria (Table 1), 1544 individuals will be selected. Participants in both arms will receive their routine care and the intervention arm will be encouraged to follow guides for each risk factor using the app in addition to the routine care. After 3 and 6 months, the status of risk factors will be determined through outpatient visits and face-to-face interviews for both arms. All the participants will be visited in an outpatient clinic by a cardiologist to evaluate the status of the risk factors and document any cardiovascular events (outcomes). Any additional visits will be considered for any patient requiring fewer interval visits.

**Fig. 1:**
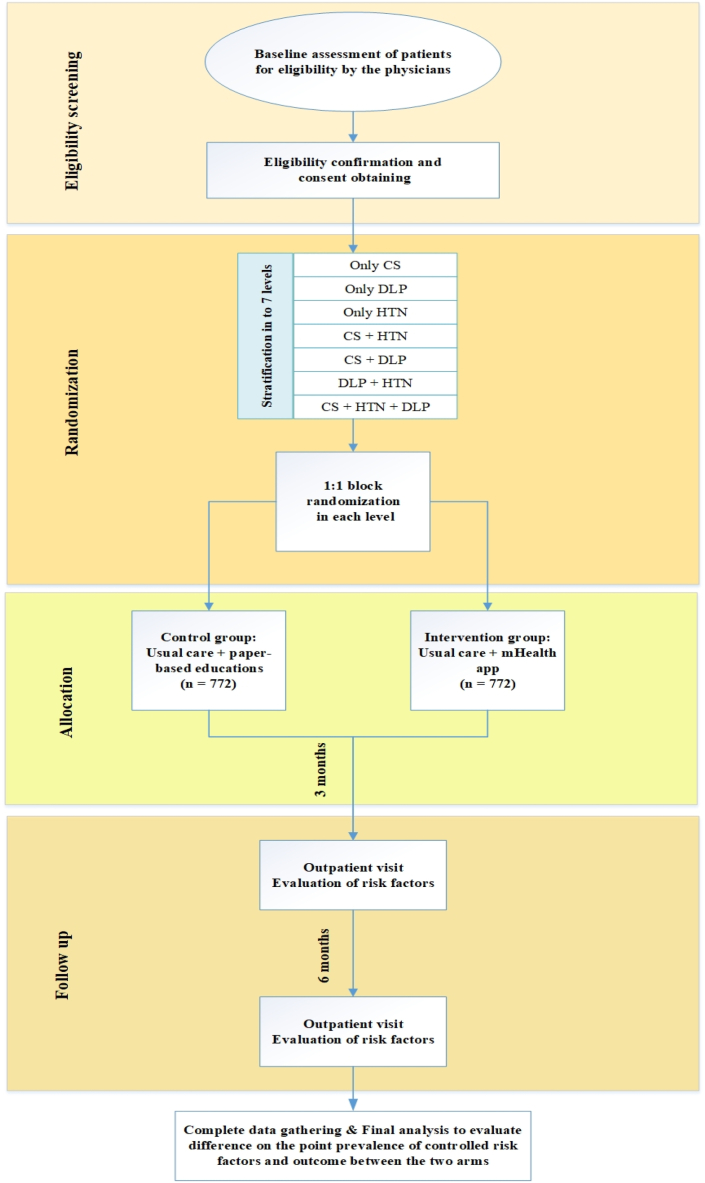
Enrollment, randomization and follow-up protocol of the study CS: Cigarette Smoking, DLP: Dyslipidemia, HTN: Hypertension.

During study time, patients in the intervention arm will be encouraged to continue to use the app. The patients in both arms will be allowed to have their routine care inside and outside THC whenever needed. The participant timeline and summary of the study protocol are summarized in Table 2 and Fig. 1, respectively.

**Table 2:** Participant timeline.

		Study period			
		Enrollment	Allocation	Post-allocation	Close-out
**Month**		−3 ± 2 days	0	3	6
**Enrollment**		×			
**Eligibility screen**		×			
**Informed consent**		×			
**Allocation**			×		
**Intervention**			×	×	×
**Assessment**	Baseline variables	×		×	×
	Measuring systolic and diastolic blood pressure	×		×	×
	Evaluating smoking status	×		×	×
	Measuring low-density lipoprotein cholesterol serum level	×		×	×
	Evaluating adherence to treatment and diet			×	×
	Evaluating the usability (data entry) and satisfaction level of software/application			×	×

### 3.9 Sample size calculation

Farkouh et al. [22] which studied pooled data obtained from large randomized trials display the success rate in controlling risk factors in CAD patients for secondary prevention through lifestyle modification and drug consumption.

To increase the success rate percentage of modifying the risk factors by at least 1.5 times, concerning significance level of α = 0.05, study power of 80% and 10% loss of samples, a sample size of 257, 538, and 772 were considered for DLP, HTN, and CS, respectively. The largest sample size of 772 which is related to CS was selected (772 × 2 = 1544). The calculation was performed by G-power software. The final results are reported in Table 3.

**Table 3:** Success rates in controlling Dyslipidemia, Hypertension and smoking and calculated sample size.

Risk Factor	Success rate percentage in the control group	Success rate percentage in the intervention group	Sample size in each group counting 10% shedding of samples	The abundance of patients referred to the Tehran Heart Center
**Dyslipidemia**	0.2	0.3	257	0.72
**Hypertension**	0.11	0.165	538	0.47
**Cigarette smoking**	0.08	0.12	772	0.34

### 3.10 Randomization and implementation

Randomization will be performed at 7 different levels based on the type and number of risk factors the patients have (stratified randomization). Participants will be categorized into the following 7 levels; 1) Only CS, 2) Only HTN, 3) Only DLP, 4) CS + HTN, 5) CS + DLP, 6) DLP + HTN, 7) CS + HTN + DLP.

Participants in each level will be randomized into two study arms as follows.

1 Intervention (case) arm: Usual care + mHealth
2 Control arm: Usual care + paper-based recommendations and educational materials

In each level, participants will be assigned into two arms by block randomization with a 1:1 allocation using variable block sizes of 2, 4, 6, 8, and 10. Allocation of each subject to the study arms will be performed online to prevent selection bias and respect allocation sequence concealment (By concealing the allocation sequence). Allocation of each participant will be performed, just after inclusion and exclusion criteria are met and informed consent is signed by the participants. Computer-generated random number list will be utilized using permuted block stratified randomization. (Block stratified randomization Windows Version 6.0, by Steven Piantadosi, M. D., Ph.D., Cedars-Sinai Medical Center).

The practitioners who evaluate the risk factors controlling status are blinded to the group assignment.

#### 3.10.1 Pilot study descriptions for mHealth app

After the development of the app, we will conduct a pilot study to evaluate the feasibility and acceptability of the app among a small sample group of volunteers in our center. The objective of this phase is to refine the design and development and pilot testing of the app. The potential participants will be recruited from THC outpatient clinics. Feasibility will be assessed by examining the participant's interactions with the app via backend reporting tools. Acceptability will be examined during follow-up visits or phone calls to examine satisfaction with the application in terms of ease of use and effectiveness. Following the pilot study, we will implement the required changes and refinements to the app before testing its efficacy in a randomized controlled trial in a larger study.

### 3.11 Data collection and statistical analysis

We are going to record all the patient's demographic and clinical characteristics.

(Including past medical history, duration of each disease, drug and habitual history, etc.) and status of their risk factors (controlled/uncontrolled). BP, height, weight, and waist circumference will be measured during each visit. The blood sample will be taken before each visit to measure the serum level of the following factors: Creatinine, LDL-C, Glycated hemoglobin (HbA1C), Total cholesterol, High-density lipoprotein (HDL-C), Fasting blood glucose (FBS) and Triglyceride. In addition, we will request all the patients to present every laboratory test report that was done even outside THC and all other documents related to any event that required hospitalization within the last year. Hypertensive patients in the control arm will receive a paper-based chart to document their BP (systolic and diastolic) for 7 consecutive days in each month (the intervention group will also enter and save the measurements in the app).

The data will be collected from a paper questionnaire which is filled out by the research team and saved information on the digital server. Data entry will be conducted on the IBM SPSS software database.

### 3.12 Statistical methods

Continuous variables will be presented through mean with the standard deviation (SD) for normally distributed variables or median with the interquartile range boundaries for skewed variables. Categorical variables will be presented as the frequency with percentage. To check the normality distribution of continuous variables, histograms will be applied along with the above descriptive quantities. Risk factors’ status will be measured at different time points to determine the final status at the end of certain time points. We will compare the prevalence of the controlled risk factors in time points of 0 (baseline), 3 and 6 months between intervention and control groups using x^2^ tests at each point. Multivariate logistic regression analysis will be used to determine the effect of the intervention on the control of risk factors at the end of 6 months. The effect will be reported as Odds Ratios with the corresponding 95% confidence interval. This will be a per-protocol approach. To perform the intention-to-treat method, participants will be analyzed according to the group they were allocated not the actual intervention they received. The missing values for subjects who were lost to follow-up or did not cooperate until the end of the study will be evaluated to see to what extent the missing data are completely random. If missing data do not violate the randomness assumption, they will be replaced using the multivariate missing imputation method. Otherwise, stratified analysis and sensitivity analysis for missing data will be considered. Finally, the size of the effect is measured and reported as a relative risk/risk ratio with a corresponding 95% confidence interval. The effect will be calculated and reported as a hazard ratio with the corresponding confidence interval.

## 4 Discussion

This clinical trial aims to assess the effectiveness of the Persian-language Green Heart application in managing key risk factors for CAD and enhancing secondary prevention strategies among the Iranian population. By utilizing mHealth technology, this research seeks to provide valuable insights into the potential benefits of digital interventions in improving cardiovascular health outcomes and reducing the burden of CAD in this specific demographic.

Although there are many evidence-based clinical practice guidelines for controlling DM, HTN management, and reduction of LDL-C, applying them to daily management is often difficult for patients and needs self-management skills. There is a lack of effective and intensive treatments for risk factors for the majority of those who are at high risk for CVD. Fundamentally, new approaches are needed to achieve optimal treatment goals for predisposing factors to CVD.

Nowadays, the use of mHealth care is becoming increasingly popular providing a good opportunity for constant monitoring of risk factors and changing health behavior in a target population. Meanwhile, providing evidence for the effectiveness of health intervention delivery using mobile technologies could help health providers encourage their at-risk population to stop smoking, control BP, control blood cholesterol and participate in regular physical activity. While the burden of CVD is growing in developing countries, this type of intervention can be a cost-effective way to reduce it in these countries, e.g., Iran. Such intervention in our country could be used for primary and secondary prevention at the population level.

The expansion of mobile healthcare led to the development of a paramount number of healthcare apps appearing on smartphones’ app stores. Despite the commercial availability of many innovative mHealth apps, few of them have been designed based on evidence-based knowledge and need careful consideration of different clinical conditions. Motivational messages, monitoring, and behavior change tools used in face-to-face support can be modified for delivery via mobile phones. Interventions can be personalized with content tailored to the age, sex, and ethnic group of the participant.

We sought to develop a health app using current clinical guidelines for controlling DLP, and HTN, which also will include smoking cessation support in patients with CAD.

## 5 Limitations

• Sometimes patients are not certain about the privacy and security of their data hence they may type or add incorrect information; we should describe the privacy protocols for them and assure them that their data is safe.
• Once the goal of the treatment is achieved; for example, BP has been controlled; there is not 100% certainty to remain controlled in the future; hence close monitoring and supervised visits are advised. However general recommendations will be given to the patients to remain healthy and have annual in-person visits.
• The benefits of “quitting smoking” or “controlling BP” may be seen years after the patient reaches these goals; the “6 months period of follow-up” maybe not be enough to measure and compare the secondary outcomes and probable events. Fortunately, in THC, a high percentage of CAD patients are followed at least annually.
• Worldwide, mHealth has been proposed to reduce in-person visits; however physical examination and drug prescription are the two mainstays of diagnosis and treatment of the risk factors. We should be alert that some patients may need in-person visits and application-based management may not suffice.

## 6 Conclusion

Developing a user-friendly application for the Iranian population will reduce costs and enhance the accessibility to healthcare facilities. Even a poor patient in a far village could have access to the best doctor in major cities and patients are not mandated to come from long distances.

The results of this study (if accepted) may help policymakers to establish country-wide mHealth technology. This app, if effective, will be used for secondary prevention across the country. In the next step, we plan to extend the contents of the app to increase the level of physical activity and decrease body weight.

## Data availability statement

The datasets generated and/or analyzed during the current study are not publicly available due to THC's policy but are available from the corresponding author upon reasonable request.

## Declarations

•This study was reviewed and approved by ethical committees of the Tehran University of Medical Sciences with the approval number: [IR.TUMS.MEDICINE.REC.1399.032]. This study was registered in the Iran Randomized Clinical Trial Center with the ID of [IRCT20221016056204N1] on March 12, 2023.
•All participants/patients (or their proxies/legal guardians) provided informed consent to participate in the study.
•Anonymised case details and images of participants will not be published in this study.

## CRediT authorship contribution statement

**Mojgan Ghavami:** Conceptualization, Data curation, Funding acquisition, Investigation, Project administration, Software, Supervision, Visualization, Writing – original draft, Writing – review & editing. **Saeed Sadeghian:** Conceptualization, Project administration. **Ayat Ahmadi:** Investigation, Methodology, Validation, Writing – review & editing. **Masoumeh Lotfi-Tokaldany:** Writing – original draft. **Mahnaz Ashoorkhani:** Software, Validation, Writing – review & editing. **Fateme Haji Ali Asgari:** Software, Visualization.

## Declaration of competing interest

The authors declare the following financial interests/personal relationships which may be considered as potential competing interests: Mojgan Ghavami reports financial support was provided by 10.13039/501100012155National Institute for Medical Research Development.
