# Supplementary material for: The effectiveness of green heart application to manage modifiable risk factors of coronary artery disease in Tehran Heart Center: Study protocol for a randomized controlled trial
Source: Heliyon. 2024 Mar 22;10(7):e28370. doi: 10.1016/j.heliyon.2024.e28370 (PMC10979141; doi:10.1016/j.heliyon.2024.e28370)
Supplement: Multimedia component 1 [file mmc1.pdf]

Clinical Trial Protocol
Iranian Registry of Clinical Trials

11 Dec 2023

The effectiveness of applying Mobile-Health Technology to manage modifiable risk factors of coronary artery disease for secondary prevention: a randomized clinical trial

Protocol summary

Study aim

This study aims to help coronary artery disease (CAD) patients in controlling three cardiovascular diseases (CVD) risk factors by applying intervention using mobile-health technology: Hypertension, Dyslipidemia, and cigarette smoking. This intervention could reduce the burden of CVD by decreasing death and disability in patients with CAD.

Design

Two-arm parallel-group single-blinded randomized controlled trial on 1544 patients. Computer-generated random number list will be utilized using permuted block stratified randomization.

Settings and conduct

Tehran Heart Center.772 patients in each arm. The practitioners who evaluate the risk factors controlling status are blinded to group assignment.

Participants/Inclusion and exclusion criteria

Inclusion criteria: History of coronary artery disease by coronary angiography confirming stenosis>50% in at least one of the major epicardial coronary arteries or their main branches, Having at least one uncontrolled risk factor including uncontrolled hypertension, uncontrolled dyslipidemia, and current cigarette smoking for ≥12 months, Aged between 25 to 75 years, Inclination to staying in study for two years, Having and ability to use a smartphone by the participant or one of his/her trustworthy relatives who live near him/her, Willing to control the risk factors using application provides informed consent to participate in the study. Exclusion criteria: Mental retardation, Individuals with prohibitive morbidity that requires close face-to-face monitoring, End-stage chronic diseases such as hepatic and/or renal failure, heart failure, active cancer

Intervention groups

1- Intervention (case) arm: Usual care + mobile-Health 2- Control arm: Usual care + paper-based education in face-to-face visits

Main outcome variables

Smoking Cessation; Blood pressure control status; Dyslipidemia control status

General information

Reason for update

Acronym

IRCT registration information

IRCT registration number: **IRCT20221016056204N1**

Registration date: **2023-03-12, 1401/12/21**

Registration timing: **retrospective**

Last update: **2023-03-12, 1401/12/21**

Update count: **0**

Registration date

2023-03-12, 1401/12/21

Registrant information

Name

Mojgan Ghavami

Name of organization / entity

Country

Iran (Islamic Republic of)

Phone

+98 21 6651 3375

Email address

m_ghavami@razi.tums.ac.ir

Recruitment status

Recruitment complete

Funding source

Expected recruitment start date

2022-11-22, 1401/09/01

Expected recruitment end date

2023-02-20, 1401/12/01

Actual recruitment start date

empty

Actual recruitment end date
empty

Trial completion date
empty

Scientific title
The effectiveness of applying Mobile-Health Technology to manage modifiable risk factors of coronary artery disease for secondary prevention: a randomized clinical trial

Public title
Effect of M-Health on secondary prevention of coronary artery disease patients

Purpose
Prevention

Inclusion/Exclusion criteria

Inclusion criteria:
History of CAD defined as history of myocardial infarction, percutaneous coronary intervention, coronary artery bypass graft surgery, coronary angiography confirming stenosis>50% in at least one of the major epicardial coronary arteries or their main branches. Having at least one uncontrolled risk factor including: uncontrolled hypertension, uncontrolled dyslipidemia and current cigarette smoking for ≥12 months. Aged between 25 to 75 years Inclination to staying in study for two years Having and ability of using smart phone by the participant or one of his/her trustworthy relatives who lives near him/her Willing to control the risk factors using App Provides informed consent to participate in the study

Exclusion criteria:
Mental retardation Individuals with prohibitive morbidity that requires close face-to-face monitoring. End stage chronic disease such as: hepatic and/or renal failure, heart failure, active cancer

Age
From **25 years** old to **75 years** old

Gender
Both

Phase
N/A

Groups that have been masked

- Investigator

Sample size
Target sample size: **1544**

Randomization (investigator's opinion)
Randomized

Randomization description
Block randomization with a 1:1 allocation using variable block sizes of 2, 4, 6, 8, and 10. Allocation of each subject to the study arms will be performed online to prevent selection bias and respect allocation sequence concealment. (By concealing the allocation sequence). Computer-generated random number list will be utilized using permuted block stratified randomization. Randomization will be performed in 7 different levels based on the type and number of risk factors the patients have. Participants will be categorized in the following 7 levels; 1 - Only CS 4 - CS + HTN 7 - CS + HTN + DLP 2 - Only HTN 5 - CS + DLP 3 - Only DLP 6 - DLP + HTN

Blinding (investigator's opinion)
Single blinded

Blinding description
The practitioners who evaluate the risk factors controlling status are blinded to group assignment.

Placebo
Not used

Assignment
Parallel

Other design features
The mHealth app asks some simple questions from each person and scores their responses. To keep close contact with participants, thereafter it gives encouraging or warning advice in short time intervals compatible with the patient’s answers. This application covers planning for smoking cessation, management of Hypertension (HTN) and Dyslipidemia (DLP). The app provides information by applying simple sentences, and educational audio and video messages about lifestyle modification. It also reminds regular time for visiting the doctor and taking drugs.

Secondary Ids

empty

Ethics committees

1

Ethics committee

Name of ethics committee
Ethics committee of Tehran University of Medical Scinces

Street address
Research institute building, Tehran Heart Center, North Kargar St

City
Tehran

Province
Tehran

Postal code
1411713138

Approval date
2020-04-30, 1399/02/11

Ethics committee reference number
IR.TUMS.MEDICINE.REC.1399.032

Health conditions studied

1

Description of health condition studied
Coronary Artery disease

ICD-10 code
125.1

ICD-10 code description
125.0

Primary outcomes

1

Description
Smoking Cessation
Timepoint
Three months post-randomization
Method of measurement
Self-report for quitting smoking

2

Description
Blood pressure control to less than 130/80 mm Hg
Timepoint
Three months post-randomization
Method of measurement
Sphygmomanometer/Blood pressure measurement by the physician in the clinic

3

Description
Dyslipidemia control with the target of low-density lipoprotein cholesterol (LDL-C) less than 55 mg/dL
Timepoint
Three months post-randomization
Method of measurement
Lab test to measure low-density lipoprotein cholesterol

Secondary outcomes

empty

Intervention groups

1

Description
Intervention group: Installation and applying mobile-health technology (Green Heart application) on the smartphone plus routine care. The Green Heart application contains sections for controlling blood pressure, dyslipidemia, and smoking cessation. Educational videos and advice are included in all sections. The blood pressure section includes "Why is blood pressure control important?", "Important points in blood pressure measurement", "Which sphygmomanometer is suitable?", "Recommendations for modifying the lifestyle of a person with hypertension", "Guide to entering maximum and minimum numbers of blood pressure based on the monitor screen of the digital device", "Guide to choosing the size of the blood pressure cuff according to the size of the arm circumference", a space for recording blood pressure where it is possible to enter information twice a day, seven days a week and with each record, the average of blood pressure recorded up to that time and its graph are displayed for the user, and a message corresponding to the average blood pressure is given about the need to visit the doctor or a warning in case of an emergency increase in blood pressure. There is also a possibility for refresh/ reinsertion of information. All the information registered by the user is stored on the server. In the dyslipidemia section, there is a video of food diet and exercise recommendations to control dyslipidemia and a space for entering the number of Low-density lipoprotein cholesterol (LDL-C) according to the picture-guide based on the patient's lab test sheet. After entering LDL-C by the user, questions about the type of dyslipidemia medication (statin/ ezetimibe) and its dose will be asked, and if the entered number is higher than the target, a corresponding message will be sent to him/her about increasing the dose of the medication or referring to the doctor to add another category of medication and if it is normal, it is recommended to continue the drug and do a lab test again after 6 months. In the smoking cessation section, the user is asked questions based on the Fagerstrom test, the duration of smoking, the frequency of quitting, the daily cost of smoking, the use of hookah, the state of the desire to smoke, and the reason for the desire to smoke. Recommendations about the side effects of hookah and the amount of recovered cost in case of complete smoking cessation are given. The degree of smoking dependence is measured, and according to that result, educational videos with the content "Why should I quit?", "How to be prepared to quit smoking?", "Things to do on the first day of quitting smoking", "Food diet and exercise recommendations while quitting smoking", "Recommendations to get rid of temptations while quitting smoking", and "Recommendations and important tips while quitting smoking" are displayed. The application is installed on the patient's mobile phone and the method of entering information and accessing different parts of the application is explained to him/her and one of his close, reliable, literate, and familiar with electronic devices companions. The user name is the mobile phone number and the password is the patient's national code, and each user's information is stored confidentially on the server. In addition to watching educational videos, the user enters information about blood pressure, dyslipidemia, and smoking status according to the instructions and guidelines.
Category
Prevention

2

Description
Control group: routine care including advice for lifestyle modification orally, in the format of booklets and pamphlets With the content of "Importance of blood pressure control", "Important points in blood pressure measurement", "Choosing the right sphygmomanometer", "Recommendations to modify the lifestyle of a person with hypertension", "Guide to entering maximum and minimum numbers of blood pressure based on the monitor screen of the digital device", "Guide to choosing the size of the blood pressure cuff according to the size of the arm circumference", "Recommendation to measure and record blood pressure twice a day for seven days on a paper blood pressure chart and bring it with themselves on the day of the visit", "Food diet and exercise

recommendations to control dyslipidemia" and a separate booklet for smokers with the content "Why should I quit?", "How to be prepared to quit smoking?", "Things to do on the first day of quitting smoking", "Diet and exercise recommendations while quitting smoking", "Recommendations to get rid of temptations while quitting smoking", and "Recommendations and important points while quitting smoking" which are presented to them.

Category
Prevention

Recruitment centers

1

Recruitment center
Name of recruitment center
Tehran Heart Center
Full name of responsible person
Mojgan Ghavami
Street address
North Kargar Ave
City
Tehran
Province
Tehran
Postal code
1411713138
Phone
+98 21 8802 9600
Fax
Email
email@thc.tums.ac.ir
Web page address
https://thc.tums.ac.ir/

Sponsors / Funding sources

1

Sponsor
Name of organization / entity
Tehran University of Medical Sciences
Full name of responsible person
Saeed Sadeghian
Street address
Corner of Qods, Keshavarz Blvd.
City
Tehran
Province
Tehran
Postal code
1417613151
Phone
+98 21 6640 5373
Email
Ssadeghian@tums.ac.ir
Grant name
Grant code / Reference number
Is the source of funding the same sponsor organization/entity?
Yes
Title of funding source
Tehran University of Medical Sciences
Proportion provided by this source
40
Public or private sector
Public
Domestic or foreign origin
Domestic
Category of foreign source of funding
empty
Country of origin
Type of organization providing the funding
Academic

Person responsible for general inquiries

Contact
Name of organization / entity
Tehran University of Medical Sciences
Full name of responsible person
Mojgan Ghavami
Position
Cardiologist
Latest degree
Specialist
Other areas of specialty/work
Cardiology
Street address
No. 5, First alley, Shafigh balkhi alley, Zolfaghari Ave, Shadmehr Ave, Sattarkhan St.
City
Tehran
Province
Tehran
Postal code
1456856553
Phone
+98 21 6651 3375
Fax
Email
m_ghavami@razi.tums.ac.ir

Person responsible for scientific inquiries

Contact
Name of organization / entity
Tehran University of Medical Sciences
Full name of responsible person
Mojgan Ghavami
Position
Cardiologist
Latest degree
Specialist
Other areas of specialty/work
Cardiology
Street address
No. 5, First alley, Shafigh balkhi alley, Zolfaghari Ave, Shadmehr Ave, Sattarkhan St.
City
Tehran

Province
Tehran
Postal code
1456856553
Phone
+98 21 6651 3375
Fax
Email
m_ghavami@razi.tums.ac.ir

Person responsible for updating data

Contact
Name of organization / entity
Tehran University of Medical Sciences
Full name of responsible person
Mojgan Ghavami
Position
Cardiologist
Latest degree
Specialist
Other areas of specialty/work
Cardiology
Street address
No. 5, First alley, Shafigh balkhi alley, Zolfaghari Ave, Shadmehr Ave, Sattarkhan St.
City
Tehran
Province
Tehran
Postal code
1456856553
Phone
+98 21 6651 3375
Fax
Email
m_ghavami@razi.tums.ac.ir

Sharing plan

Deidentified Individual Participant Data Set (IPD)
Undecided - It is not yet known if there will be a plan to make this available
Study Protocol
Undecided - It is not yet known if there will be a plan to make this available
Statistical Analysis Plan
Undecided - It is not yet known if there will be a plan to make this available
Informed Consent Form
Undecided - It is not yet known if there will be a plan to make this available
Clinical Study Report
Undecided - It is not yet known if there will be a plan to make this available
Analytic Code
Undecided - It is not yet known if there will be a plan to make this available
Data Dictionary
Undecided - It is not yet known if there will be a plan to make this available
