# Supplementary material for: The effectiveness of green heart application to manage modifiable risk factors of coronary artery disease in Tehran Heart Center: Study protocol for a randomized controlled trial
Source: Heliyon. 2024 Mar 22;10(7):e28370. doi: 10.1016/j.heliyon.2024.e28370 (PMC10979141; doi:10.1016/j.heliyon.2024.e28370)
Supplement: Multimedia component 2 [file mmc2.docx]

**Declaration of interests**
 
☐ The authors declare that they have no known competing financial interests or personal relationships that could have appeared to influence the work reported in this paper.
 
☒ The authors declare the following financial interests which may be considered as potential competing interests:

| Mojgan Ghavami reports financial support was provided by National Institute for Medical Research Development with **grant number 4001391** with no role in study design, data collection, analysis, interpretation, writing, and submission of the manuscript. This support did not affect the authors’ objectivity and did not influence the content of the article. |
| --- |

**There are no professional relationships and no patents to disclose.**

**There are no additional relationships or activities to declare.**
